# Area-Level Socioeconomic Status, Adiposity, Physical Activity, and Inflammation in Young Adults, 2013

**DOI:** 10.5888/pcd11.140090

**Published:** 2014-07-31

**Authors:** Michael V. Fedewa, Bhibha M. Das, Ronald L. Forehand, Ellen M. Evans

**Affiliations:** Author Affiliations: Bhibha M. Das, East Carolina University, Greenville, North Carolina; Ronald L. Forehand, Ellen M. Evans, University of Georgia, Athens, Georgia.

## Abstract

**Introduction:**

We assessed the independent effects of socioeconomic status, sex, adiposity, and physical activity on C-reactive protein in young adults.

**Methods:**

During the fall semester of their first year, college students (n = 177; mean age, 18.1 y; 66.7% female; 65.5% white) were assessed for adiposity via dual x-ray absorptiometry, physical activity via accelerometer, and serum C-reactive protein. Area-level socioeconomic status was based on self-reported home zip code. Hierarchical linear modeling was used to assess the relationship of sex, adiposity, and physical activity on the dependent variable of C-reactive protein, with participants nested within geographic regions of similar socioeconomic characteristics.

**Results:**

C-reactive protein was positively associated with adiposity and inversely associated with socioeconomic status (both *P* < .05). Area-level socioeconomic status explained 28.2% of the variance in C-reactive protein. Adiposity was significantly associated with C-reactive protein in the full model (*P* = .006); physical activity was not associated with C-reactive protein (*P* = .48), and area-level socioeconomic status approached significance (*P* = .05) within the age range of our analysis after accounting for the variance explained by adiposity.

**Conclusion:**

The significant positive association between adiposity and C-reactive protein suggests that young adults with higher adiposity have higher C-reactive protein levels after accounting for area-level socioeconomic status, sex, and physical activity.

## Introduction

Cardiovascular disease (CVD) is the leading cause of death in the United States, totaling nearly 600,000 deaths in 2010 (1). Although age is a key factor in CVD, increased focus is on the prevention of risk factors, or primordial prevention. CVD risk factors may appear in young adults long before overt disease manifests. Prevention of CVD involves adopting numerous health-related behaviors aimed at reducing and controlling traditional disease risk factors (eg, hypertension, dyslipidemia, obesity). In addition to the traditional risk factors, high sensitivity C-reactive protein (CRP) is a marker of chronic systemic inflammation that has emerged as a strong independent risk factor of future CVD (2–5).

Identifying underlying factors that may influence CRP levels becomes important given the strong association with future disease risk. CRP levels are positively associated with relative adiposity (%FAT) in healthy people, and obese men and women are twice as likely to have elevated CRP levels than adults of normal weight (6,7). In addition, regular moderate- and vigorous-intensity physical activity (PA) is negatively associated with CRP and may indirectly affect CRP by favorably influencing %FAT (8,9).

Low socioeconomic status (SES) is linked with adverse health behaviors, such as physical inactivity and poor diet, and negative health outcomes including obesity (10–12). A systematic review of 333 published studies found a negative relationship in 63% of effects measuring the association between SES and body size among women (13). In a cross-sectional study of more than 6,000 men and women in the United States, wages were negatively associated with body mass index (BMI) and obesity, suggesting that adults with lower wages have higher BMI and are more likely to be obese or vice versa (14). An emerging body of research demonstrates that unhealthy neighborhood-level environmental factors, including lack of PA resources and healthful food options, are associated with higher rates of obesity and other poor health indicators, and such environments may lead to lower levels of PA (15,16). In a cross-sectional analysis of 284 Mexican American women, an increase of 1 standard deviation in SES was associated with a 27% decrease in CRP (17). A prospective study of more than 6,000 European adults found lower-SES adults were 1.61 times more likely to have high CRP than their more affluent peers and were more likely to have higher CRP over a 16-year period (18). In a prospective analysis of 3,330 young adults (mean age, 32 y), participants in the lowest SES group were nearly 10 times more likely to have multiple elevated CRP measurements over a 13-year period; the study also found that SES may influence CRP indirectly by affecting %FAT, diet, and habitual PA (19,20).

The aims of our study were to assess the independent and interactive effects of SES, %FAT, and PA on CRP in young adults. It was hypothesized that 1) first-year college students from lower SES areas will have higher CRP levels, 2) %FAT will be positively associated with CRP levels, and 3) PA and CRP will be negatively associated.

## Methods

First-year college students (n = 177) were recruited via email and print advertising during the fall semester of 2013 at the University of Georgia. E-mail messages were sent directly to the e-mail account created by the university for each student upon enrollment, through a listserve created by the Office of the Registrar. Participants were required to be full-time, first-year students aged 18 to 20 years residing on campus. Varsity athletes were excluded, as were participants who were pregnant, planning to become pregnant, or had given birth in the previous 12 months. This study protocol and informed consent document were approved by the Institutional Review Board at the University of Georgia.

Participants completed 2 visits to the measurement laboratory, and visits were completed 8 days apart to allow for PA monitoring in the interim. Informed consent was completed at the initial visit. Participants completed basic demographic questionnaires online between visits. Anthropometric measures were collected at the second visit. Standing height was measured by a stadiometer (Seca 242, SECA Corp, Hamburg, Germany) to the nearest 0.1 cm. Weight was measured with a digital scale (Tanita WB-110A class III, Tanita Corporation, Tokyo, Japan) to the nearest 0.1 kg. BMI was calculated as weight divided by height squared (kg/m^2^). %FAT was measured via dual x-ray absorptiometry (Lunar iDXA, v 11.30.062, GE Healthcare, Madison, Wisconsin). Objective PA was measured in steps per day over 8 consecutive days using the NL-1000 accelerometer (New-Lifestyles Inc, Lee’s Summit, Missouri). Participants with fewer than 4 valid days with at least 10 hours of valid wear time were excluded from the analysis. CRP was measured after a 12-hour fast by using conventional clinical methods and was reported in mg/L. CRP levels were dichotomized into categories representing elevated (>3 mg/L) and normal (<3 mg/L) (5). Patients with fasting glucose greater than 126 mg/dL, indicating possible diabetes, were excluded.

Area-level SES was estimated using the Townsend Index (TI) of material deprivation (21). Self-reported home postal zip code was used as a proxy for an individual’s area to classify each participant based on SES. The TI is a measure of SES of a geographic region consisting of a standardized *z* score combining data on percentage of household crowding, unemployment rate, percentage of individuals with no car, and percentage of households not owner-occupied in a given area. Data on area unemployment rate, home ownership rate, car ownership rate, and household overcrowding for each zip code were obtained from the 2010 American Community Survey (22). Negative values of the overall TI scores reflect less deprived areas; positive values reflect more deprived areas. The 4 variables — area unemployment, home ownership, car ownership, and household overcrowding — combine to form an overall score.

Descriptive statistics and bivariate correlations were calculated using Statistical Package for the Social Sciences analysis software (SPSS, Inc, version 20.0 for Windows, Chicago, Illinois). All data expressed are in mean (standard deviation [SD]). In addition, hierarchical linear modeling (HLM) was used in this cross-sectional analysis to establish the overall magnitude and significance of the relationship between CRP, %FAT, PA, and SES using HLM 7 (Scientific Software International, Inc. version 7 for Windows, Skokie, Illinois). HLM allowed for the analysis of the relationship between dependent (CRP) and independent (%FAT, PA, SES) variables when the same zip code was represented more than once in the data set by adjusting for the nonindependence or nesting of these values. For each model, zip code was included to identify nesting of scores from the geographic area. After calculating the TI for each participant, cases were collapsed across zip codes to create 30 groups of similar SES characteristics with a minimum of 3 cases per group. Cases with missing data were excluded from the analysis. Significance was indicated using an α level of *P* < .05.

### Analytic rationale

Although sex was not a primary variable of interest, it was included in the model because of the biological differences in %FAT between sexes. Using a 2-level HLM allows for the analysis of the dependent variable (CRP) and level-1 independent variables (sex, %FAT, PA) when participants can be nested within level-2 geographic regions of similar economic characteristics to adjust for nonindependence of the results. For each model, an area-level SES variable was included to identify nesting of scores from areas with similar SES characteristics.

The unconditional means model tested whether the intercept of log CRP differed by SES group. The best fitting model was determined (random intercept or random slope) and that model was used in subsequent analyses. The fully unconditional model did not specify any predictors at either level 1 or 2. The purpose of the subsequent conditional models was to add predictors to the final unconditional model and determine their significance. The additional level-1 predictors included in the conditional models are %FAT, sex, and PA. Area-level SES was the only additional level-2 predictor included in the conditional models. Because a step count equal to zero is not a value that even the most sedentary individuals would expect to record, and because a %FAT measure of zero is biologically implausible, %FAT and PA were added to the model centered on the grand mean of the parameter to enhance interpretation. Further analysis was performed to determine the effect of additional level-2 variables that may affect mean log CRP. In the second model, area-level SES was added as a level-2 variable to determine whether area-level SES is associated with log CRP. In our study, outliers (>3 SD) were removed from the data, and log transformation of CRP was used to reduce skewness and kurtosis.

## Results

We had 177 level-1 cases (mean [SD] age = 18.1 [0.3] y, 66.7% female, 65.5% white) nested in 30 level-2 geographic regions (mean 6.0 [1.2] cases per group) (Table 1). The number of cases per zip code ranged from 1 to 5 (mean 1.4 [0.9] cases per zip code), indicating that our sample population was gathered from diverse geographic regions. TI scores ranged from −7.86 to 8.98 (mean 0.00 [3.09]). Although most (43.4%) students reported annual household income from $50,000 to $99,999, 13.3% of the sample reported annual household income below $35,000, with 7.9% reporting reliance on some form of government assistance to supplement monthly household income. A sample correlation matrix is provided in Table 2, after controlling for the influence of sex. Twenty-two students in our sample had elevated CRP levels.

**Table 1:** Sample Characteristics of First-Year College Students by Sex^a^ (n = 177), 2013

Characteristic	Female, Mean (Standard Deviation)	Male, Mean (Standard Deviation)	Total, Mean (Standard Deviation)
Age, y	18.1 (0.3)	18.1 (0.4)	18.1 (0.3)
Body mass index, kg/m^2^	23.1 (3.7)	22.9 (3.5)	23.0 (3.6)
%FAT	32.9 (5.4)	19.9 (5.9)	28.5 (8.3)
C-reactive protein, mg/L	1.3 (1.8)	1.4 (2.4)	1.3 (2.0)
Physical activity^b^	11,529.4 (2,851.1)	11,803.8 (2,653.2)	11,620.8 (2,782.2)

Abbreviation: %FAT, relative adiposity measured via dual x-ray absorptiometry.

a Sex determined via self-report as female or male.

b Measured as steps per day via accelerometer.

**Table 2:** Correlation Matrix of C-Reactive Protein, %FAT, Physical Activity, and Socioeconomic Status Controlling for Sex Among First-Year College Students (n = 177), 2013

Variable	Log CRP	%FAT	Physical Activity^a^	Socioeconomic Status^b^
Log CRP	1.00	—	—	—
%FAT	.25^c^	1.00	—	—
Physical activity	−.08	−.24^c^	1.00	—
Socioeconomic status	.19^d^	.09	.04	1.00

Abbreviations: log CRP, transformed measure of C-reactive protein; %FAT, relative adiposity measured via dual x-ray absorptiometry; —, not included.

a Measured via accelerometer and reported as steps per day divided by 1,000 so that all variables are expressed on a similar scale.

b Determined by using the Townsend Index of material deprivation (21).

c *P* < .001.

d *P* = .01.

### Unconditional models

The intraclass correlation coefficient for log CRP and area-level SES was equal to 13.8% and justified the use of a hierarchical modeling approach because the intraclass correlation coefficient exceeded 10% (23).

A 2-level hierarchical model examined the effect of geographic area on log CRP with geographic area entered as a random effect. On the basis of our sample of young adults, log CRP varied significantly between geographic regions, and the positive association between SES and log CRP suggested that students from lower SES areas (indicated by a higher TI) had higher CRP levels (*u_0j_* = 0.05, *P* < .001). The proportion of variance explained by area-level SES was 28.2%.

### Conditional models

The second model converged as well, because area-level SES was significantly positively associated with log CRP (*t*[28] = 2.17, *P* = .04). The addition of sex, %FAT, and PA increased amount of variation explained in each subsequent model by 1.40%, 4.12%, and 0.59%, respectively. Only %FAT was positively associated with log CRP in the final conditional model. The significant and positive association between %FAT and log CRP suggests that young adults with higher %FAT (β = 2.15, *P* = .006) have higher log CRP levels after accounting for area-level SES, sex, and PA. PA and sex were not associated with log CRP within the age range of our analysis after accounting for %FAT. Finally, area-level SES was not significantly associated with log CRP in young healthy adults after accounting for sex, %FAT, and PA (β = 2.15, *P* = .05).

Model A represents the 1-way random effect analysis of variance model with a grand mean γ_00_, a group effect *u_0j_*, and a person effect *r_ij_*. Model E represents the full model with all potential moderators included. Level-1 fixed effects were removed to create reduced models nested within the full model beginning with PA to create model D. Model D did not provide a significantly worse fit when compared with model E (*P* = .48). Removing %FAT in addition to PA to create model C yielded a model fit that was significantly worse than the full model (Model E) (*P* = .01). Finally, model B provided a significantly worse fit when compared with the full model (*P* = .01); however, it was no different than model C (*P* = .26). Parameter estimates for each model along with the coefficient and SE for each parameter in the full model are provided in Table 3.

**Table 3:** Hierarchical Linear Model Results Predicting C-Reactive Protein From %FAT, Physical Activity, Socioeconomic Status (SES), and Sex Among First-Year College Students (n = 177), 2013

Fixed Effects	Parameter	Log CRP					*P* Value^f^
		Model A^a^	Model B^b^	Model C^c^	Model D^d^	Model E, β (SE)^e^	
**Composite model**							
Intercept	γ_00_	−0.26	−0.26	−0.26	−0.32	−0.32 (0.07)	<.001
SES	γ_01_	—	0.04	0.04	0.03	0.03 (0.02)	.05
Sex^g^	γ_10_	—	—	0.10	0.18	0.18 (0.13)	.17
%FAT	γ_20_	—	—	—	2.28	2.15 (0.75)	.006
Physical activity^h^	γ_30_	—	—	—	—	−0.01 (0.15)	.48
**Goodness of fit, deviance**	NA	303.46	299.11	297.83	288.39	287.89	NA

Abbreviations: %FAT, relative adiposity measured by dual x-ray absorptiometry; SE, standard error; —, not included in model; NA, not applicable.

a Analysis of variance model to detect differences between SES groups.

b Area-level SES included as a level-2 predictor.

c Sex included as level-1 predictor, area-level SES included as a level-2 predictor.

d Sex and %FAT included as level-1 predictors, area-level SES included as a level-2 predictor.

e Full model with sex, %FAT, and physical activity included as level-1 predictors, area-level SES included as a level-2 predictor.

f Calculated by using χ^2^ distribution with degrees of freedom equal to the number of parameters estimated.

g Sex classified as female or male (coded 0 or 1, respectively).

h Measured via accelerometer and reported as steps per day divided by 1,000 so that all variables are expressed on a similar scale.

## Discussion

The results of this study provide an estimate of the independent and interactive associations of SES, sex, %FAT, and PA on CRP in first-year college students. Strengths of the study include objective measures of %FAT and PA, which are not subject to the same reporting bias or social desirability influence as subjective self-reported measures. The PA level of first-year college students measured in steps per day appears to be slightly higher than estimates of the average PA level in US adults from National Health and Nutrition Examination Survey (NHANES) 2005 through 2006 and similar to previous estimates of PA in college students (24,25). An additional strength of this study is using an objective criterion measure of %FAT to measure the association between obesity and SES, which has not yet been examined in depth. The results of our study indicate that CRP levels are associated with SES in first-year college students, and %FAT is positively associated with CRP after accounting for the variation explained by SES, PA, and sex.

The association between SES and log CRP (*r* = .19, *P* = .01) indicates that first-year college students from lower SES areas have higher CRP levels when compared with their more affluent peers and supports the initial hypothesis. Because age is commonly associated with CRP levels, the association between SES and CRP in our study is novel, because it is found in a young cohort compared with previous studies linking SES and CRP (26,27). In a European cohort of 6,387 participants (aged 35–55 y at baseline, 28.5% female), adults with lower SES were 1.6 times more likely to have elevated CRP (18). Results from NHANES found an inverse relationship between CRP and SES in 6,946 adults in the United States, with CRP levels higher in adults living in poverty (mean age 42.4 [18.0] y, 58.0% female) compared with adults above the poverty threshold (mean age 46.5 [18.6] y, 49.3% female). A more pronounced difference was observed among adults with very high CRP levels (>10 mg/L), with a prevalence of 15.7% and 9.1% in adults below and above the poverty threshold, respectively (28). A prospective cohort study of 2,658 young adults found a smaller increase in CRP levels among adults with higher education and higher household income, supporting the belief that SES may mediate the change in CRP through other health behaviors such as smoking, PA, and the consumption of fruits and vegetables (20). Our study adds to the body of literature by providing evidence supporting the relationship between CRP and SES in a cohort of adults younger than participants in previously published literature and by providing objective measures of %FAT and PA.

Although our study supports the associations of SES, %FAT, and PA on log CRP in young adults, it is not without potential limitations. First, the cross-sectional nature of the study provides 1 measurement for each individual on a single occasion and cannot estimate how individuals change over time. The causal role of low PA influencing %FAT and inflammation in our study can only be hypothesized. In addition, although area-level SES could be estimated based on the zip code, no information was gathered for the length of time each participant lived at their current address. A lifetime of economic hardship may confer negative health consequences; however, the lack of information related to the duration of residence in a given area highlights an additional weakness of using area-level SES. Thus, it is assumed that each individual has lived in areas with similar area-level SES and assumes the material deprivation experienced by living in these areas provides a great enough exposure to lead to negative health consequences at a young age.

The area-level SES was estimated based on the home address of each participant, not the campus address. First-year students are required to live on campus during their first year, and although their zip code changes with the move to campus, their SES does not. In addition, all measures were obtained within 12 weeks of moving to campus, and the potential effect of changing an address is not assumed to drastically alter PA, %FAT, or CRP. Our study did not examine the potential influence of diet on CRP. Although the relationship between poor diet and increased risk of disease development has been established, examining the potential influence of poor nutritional choices on CRP in a healthy college population was beyond the scope of our study. Finally, the percentage of students receiving need-based financial aid and the total amount of financial aid received by each student was not measured in our study. Measuring the number of students receiving need-based financial aid would provide additional information that would allow us to further characterize the SES of our participants. In addition to data on household income and supplemental government assistance, financial aid information should be obtained in future research involving SES and college-age participants.

%FAT was significantly associated with log CRP; SES, sex, and PA were not associated with log CRP in our cohort of young adults, although SES approached significance. The significant positive association between %FAT and log CRP suggests that young adults with higher %FAT have higher log CRP levels after accounting for SES, sex, and PA. Although not significant in the final model, our data suggest SES may influence CRP levels in young adults. Future research should examine the potential influence of diet on CRP and the interactive effect of PA and diet on %FAT and CRP. Our study may have substantial public health importance because understanding the relationship between CRP, %FAT, and area-level SES will provide practitioners with knowledge for health interventions targeted to lower-SES populations. Primordial prevention of CVD is of utmost public health interest. Development and implementation of health promotion programs targeted at college students from disadvantaged areas may provide the opportunity for meaningful behavior change that can aid in the prevention of CVD.
